# Arteriovenous specification: BMPER and TWSG1 determine endothelial cell fate via activation of synergistic BMP and Notch signaling

**DOI:** 10.1111/febs.14439

**Published:** 2018-04-14

**Authors:** Selma Osmanagic‐Myers, Günther A. Rezniczek

**Affiliations:** ^1^ Department of Biotechnology BOKU – University of Natural Resources and Life Sciences Vienna Austria; ^2^ Department of Obstetrics and Gynecology Ruhr‐Universität Bochum Herne Germany

## Abstract

Two extracellular BMP modulators, BMPER and TWSG1, act in a pro‐BMP fashion to activate endothelial‐specific members of the TGF‐β/BMP receptor family. Through cross‐talk with the Notch signaling pathways, they are key regulators of downstream Notch targets, including ephrin B2. This adds to our understanding of BMP and Notch signaling, how these pathways converge, and thereby control arteriovenous specification.

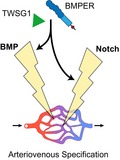

**Abbreviations**
ALK: Activin receptor‐like kinase
AVM: arteriovenous malformations
BMP: bone morphogenetic protein
BMPER: BMP endothelial precursor‐derived regulator
CoA: coactivator
DLL4: delta‐like protein 4
FGF: fibroblast growth factor
HES: hairy and enhancer of split
HEY: hairy/enhancer‐of‐split‐related with YRPW motif
TWSG1: twisted gastrulation protein homolog 1
VEGF: vascular endothelial growth factor

The complex hierarchical structure of the vascular tree requires precise spatio‐temporal control of cell fate decisions during development. These decisions rely on the selective and coordinated microenvironment‐dependent activation of a number of signaling cascades, such as the Notch, Hedgehog, vascular endothelial growth factor (VEGF), and transforming growth factor beta (TGFβ) pathways 1. During embryonic development, formation of blood vessel networks relies on two processes: vasculogenesis, the *de novo* formation of blood vessels from differentiation of mesodermal precursor cells, and angiogenesis, the expansion of a pre‐existing vascular network through sprouting or splitting of vessels 2. The arterial and venous identities are determined by the mutual ligands/receptors ephrin B2 and EphB4, respectively, which regulate repulsion and segregation of endothelial cells 3. Notch signaling controls, in particular, arterial specification. Activation of Notch is triggered by binding of the cell surface ligands, delta‐like protein 4 (DLL4) or Jagged, expressed either on endothelial cells (homotypic signaling) or smooth muscle cells (heterotypic signaling), which leads to the proteolytic cleavage and translocation of its intracellular domain (NICD) to the nucleus. There, it is recruited to target genes by binding to RBPJκ (Recombination signal‐Binding Protein for immunoglobin‐κ J region; also known as CSL) and enlistment of additional transcriptional activators 1, resulting in the expression of Notch target genes such as the HES (hairy and enhancer of split) and HEY (hairy/enhancer‐of‐split‐related with YRPW motif) families of transcriptional suppressors, but importantly also of the arterial marker ephrin B2 (Fig. 1). BMP (bone morphogenetic protein) is one of the extracellular factors that acts in cooperation with Notch in the arterial endothelium. BMP mediates activation of ALK (Activin receptor‐like kinase, an endothelial‐specific member of the TGF‐β/BMP receptor family) and the subsequent phosphorylation of SMADs. The cross‐talk between the Notch and TGF‐β/BMP pathways occurs at the level of SMADs that form a complex with NICD in the nucleus and potentiate HES/HEY gene expression 2. The outcome of Notch signaling highly depends on the particular cellular context, including the presence of extracellular proteins in the microenvironment at certain concentrations. These include BMP and typical modulators of BMP such as TWSG1 (twisted gastrulation protein homolog 1), chordin, and BMPER (BMP endothelial precursor‐derived regulator) 4, 5.

**Figure 1:**
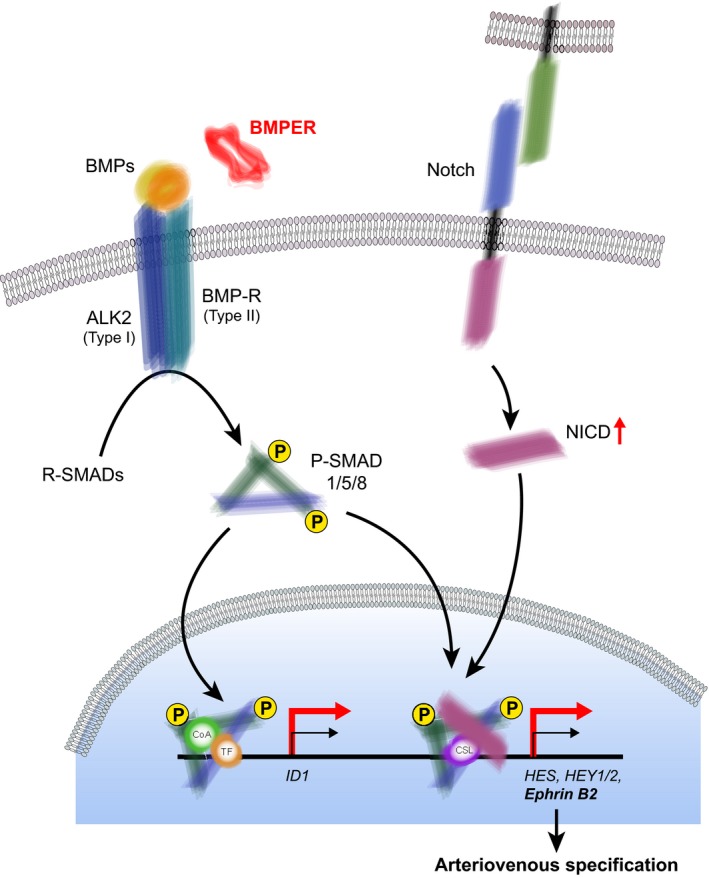
Simplified schematic model of BMP and Notch signaling activation in endothelial cells through the extracellular BMP modulators BMPER and TWSG 1. Binding of BMPs to their receptors triggers phosphorylation of SMADs which translocate to the nucleus and recruit different coactivators and transcription factors. This leads to the activation of BMP target genes such as ID1. The activation of Notch is triggered by its binding to cell surface ligands such as delta‐like protein 4 (DLL4) or Jagged exposed by adjacent cells and subsequent proteolytic cleavage and translocation of its intracellular domain (NICD) to the nucleus. Cross‐talk occurs at the level of coactivator BMP‐SMADs which are recruited to NICD bound to the Notch target gene DNA‐binding protein CSL (also known as Recombination signal‐Binding Protein for immunoglobin‐κ J region, RBPJκ). This in turn leads to activation of Notch target genes such as HES and HEY1/2, and importantly also to the expression of the arterial marker ephrin B2 (reviewed in 2). BMPER (and TWSG1) increase the expression of major BMP and Notch signaling targets (indicated by the thick red transcription indicators). ALK, activin receptor‐like kinase; BMP, bone morphogenetic protein; BMPER, BMP endothelial precursor‐derived regulator; BMP‐R, BMP receptor; CoA, coactivator; CSL, transcriptional regulator RBPJκ; HES, hairy and enhancer of split; HEY, hairy/enhancer‐of‐split related with YRPW motif; NICD, Notch intracellular domain; P‐SMAD, activated SMAD; R‐SMAD, receptor SMAD, TF, transcription factor.

In this issue of *The FEBS Journal*, Esser *et al*. decipher the role of two BMP modulators, BMPER and TWSG1, and their impact on arteriovenous specification of the endothelium via Notch signaling pathway cross‐talk 6. BMPER, originally identified in a screen for differentially expressed proteins in embryonic endothelial precursor cells, had previously been shown to exert both, anti‐ and pro‐BMP activities 4, 7. This apparent contradiction might reflect not only tissue‐specific but also BMPER concentration‐specific effects. Indeed, pro‐BMP function was observed at lower BMPER concentrations, whereas higher BMPER concentrations led to diminished BMP4‐receptor binding and endocytosis of BMP‐4/BMPER complexes 8. Esser *et al*. now demonstrate that, in endothelial cells, BMPER acts in a pro‐BMP fashion through ALK2 activation and subsequent increase in expression of its targets, such as ID1 (Fig. 1; thick red arrows). Consistent with BMP/Notch signaling cross‐talk, BMPER (and also TWSG1) caused activation of Notch, as demonstrated by increased levels of NICD, and activation of all major Notch downstream targets (HES, HEY1/2; Fig. 1; thick red arrow). As Esser *et al*. nicely demonstrate, this cross‐talk occurs intracellularly, possibly similarly to BMP‐4 effects, at the level of SMADs, and not due to direct extracellular effects on the Notch receptor, since application of a specific ALK2 inhibitor, DMH1, completely abolishes BMPER's effect on Notch target activation.

Previous reports have shown a direct interaction of BMPER and BMP‐4 7. Both proteins were shown to activate endothelial cell sprouting and migration in a dose‐dependent manner and to generate a proangiogenic response 4. Importantly, BMPER's dependence on BMP‐4 and vice versa indicated a synergistic action of both proteins. On the mechanistic level, BMPER induced the activation of SMADs 1/5 and the noncanonical Erk1/2 signaling pathway 4. Heinke *et al*. suggested that BMPER potentiates BMP delivery to its receptor, contributing to BMP gradient formation and signal sharpening, similar to the function of the BMPER homolog crossveinless‐2 in *Drosophila* 9. The study by Esser *et al*. in this issue of *The FEBS Journal* significantly widens our current perspective on BMPER function and solves part of the BMP signaling puzzle by uncovering its cross‐talk with the Notch pathway and activation of Notch downstream targets. Whether this cross‐talk happens at the level of SMADs and/or at other steps of the signaling cascades will need to be clarified in future studies. Furthermore, the integration of this synergistic Notch/BMP pathway and cross‐talk with other known BMPER‐dependent signaling pathways also still requires further elucidation. For instance, in a recent study, the proangiogenic effect of BMPER was attributed to FGF (fibroblast growth factor) signaling pathway activation 10. Additionally, atheroprotective and anti‐inflammatory actions of BMPER have been described, where BMPER acted as a BMP‐2 antagonist and affected all major targets involved in atherosclerosis development, such as atheroprotective eNOS and the proinflammatory adhesive molecules VCAM and ICAM 11. Thus, the wide range of ligands interacting with BMPER in the extracellular space, together with their context‐ and concentration‐dependent effects, further add to the cross‐talk complexity of BMPER action.

The significant finding of the study by Esser *et al*. is that BMPER and TWSG1, through upregulation of Notch pathway downstream targets, activate the expression of the gene encoding the arterial marker ephrin B2 (Fig. 1; thick red arrows). Accordingly, silencing of BMPER and TWSG1 by morpholinos in zebrafish embryos, resulted in aberrant arteriovenous specification as shown by increased *efnb4* and deregulated *efnb2b* expression, the presence of arteriovenous shunts, and impaired blood flow. In mouse embryos, similar phenotypes of arteriovenous malformations (AVM) were reported in case of mutations in the Notch ligand DLL4, the Notch signaling transcription factor CSL, as well as the Notch targets HEY1/2, with additionally upregulated venous marker expression in case of mutated DLL4 5, 12, 13. The strong phenotypic correlation of the BMPER and TWSG1 effects described by Esser *et al*. with those observed in the mutants described above, further supports the notion that BMPER and TWSG1 regulate Notch signaling also *in vivo*. Additionally, the ALK2 inhibitor DMH1 induced the same phenotype in zebrafish embryos, confirming a pro‐BMP mode of BMPER action *in vivo* 6.

In summary, the work of Esser *et al*. identifies two extracellular BMP modulators, BMPER and TWSG1, as key regulators of Notch signaling. Their action on the Notch pathway, mediated through cross‐talk with the BMP pathway, is crucial for arteriovenous specification. These results add to our understanding of the mechanisms that regulate BMP and Notch signaling, how these pathways converge and thus mediate arteriovenous specification. This is of crucial importance for the development of therapeutic or preventive approaches targeting AVM pathologies, which currently are still elusive 14.
